# Effects of Traditional Chinese Medicine for Prevention and Management of Dry Eye Disease: A Systematic Review, Network Meta-Analysis and Meta-Regression of Randomized Controlled Trials

**DOI:** 10.3390/vision10040076

**Published:** 2026-10-06

**Authors:** Weili Hu, Yuqing Jiang, Man Sau Wong, Liping Zhou

**Affiliations:** 1 Centre for Eye and Vision Research (CEVR), 17W Hong Kong, Science Park, Hong Kong; weili.hu@gmail.com (W.H.); jyqing666@gmail.com (Y.J.); 2 Department of Food Science and Nutrition, The Hong Kong Polytechnic University, Kowloon, Hong Kong; 3 Research Centre for Chinese Medicine Innovation, The Hong Kong Polytechnic University, Kowloon, Hong Kong

**Keywords:** traditional Chinese medicine, dry eye disease, tear break up time, network meta-analysis, meta-regression

## Abstract

Dry eye disease (DED) is a prevalent ocular surface disorder with diverse clinical presentations, yet the comparative efficacy of various Traditional Chinese Medicine (TCM) formulations remains unclear. This systematic review and network meta-analysis evaluated the comparative effects and baseline response relationships of TCM classes for DED across 33 randomized controlled trials retrieved from PubMed, Embase, Cochrane Library, CINAHL, and CNKI. Primary outcomes included Schirmer I test (SIT), tear film break-up time (TBUT), and corneal fluorescein staining (FL). Random-effects models estimated weighted mean differences (WMD) with 95% credible intervals (CrIs); for FL, which was measured on heterogeneous grading scales and standardized mean differences (SMD) were used. TCM classes exhibited outcome-specific profiles: Yin–Kidney (YK) and Qi–Yin Support (QYS) produced the greatest improvements in SIT versus standard care (YK: WMD 2.56, 95% CrI 0.25 to 4.86; QYS: WMD 5.32, 95% CrI 0.63 to 10.06) and significantly improved TBUT (YK: WMD 2.04, 95% CrI 1.12 to 2.89; YM: WMD 2.49, 95% CrI 0.64 to 4.37). YK demonstrated the greatest FL reduction (SMD −0.37, 95% CrI −0.71 to 0.00), followed by Liver–Spleen Regulating (SMD −0.30, 95% CrI −0.99 to 0.39). Meta-regression revealed that patients with more severe baseline deficits in tear secretion (lower SIT) and more extensive ocular-surface damage (higher FL) achieved greater clinical improvements, whereas baseline of TBUT did not significantly moderate treatment response. Overall, TCM serves as an effective adjunctive therapy for DED, with selection guided by clinical pattern and target outcomes.

## 1. Introduction

Dry eye disease (DED) reflects the multifactorial failure of tear-film homeostasis, manifesting as instability, inflammation, and neurosensory dysfunction. It can affect up to half of individuals across studies, resulting in substantial quality-of-life and work-productivity burdens [1,2]. In light of this, traditional Chinese medicine (TCM) offers pattern-based therapeutic strategies that align with common DED presentations: Liver–Kidney Yin deficiency, Liver-Qi stagnation with Spleen weakness, Qi–Yin dual deficiency with concomitant heat/blood stasis, and Lung–Stomach Yin dryness, a taxonomy reflected in clinical research and practice, from registered trials to syndrome-distribution studies and constitution analyses in dry-eye cohorts [3,4,5]. Across randomized studies and systematic syntheses, TCM has been associated with improved DED endpoints, such as extended tear film break-up time (TBUT), increased Schirmer I test (SIT) values, and decreased corneal fluorescein staining (FL). TCM is often tested in adjunctive designs (oral TCM with artificial tears vs. placebo with artificial tears) that cleanly estimate the extra effect attributable to TCM [6]. Lycium–Rehmannia pills (Qi Ju Di Huang Wan), classically used to nourish Liver–Kidney Yin for eye disorders, map directly to the Liver–Kidney Yin deficiency pattern, and a meta-analysis of randomized trials found that adding this formula to conventional therapy improved SIT and TBUT versus conventional therapy alone; earlier randomized work with Chi-Ju-Di-Huang-Wan reported symptom and staining benefits, reinforcing this link [7,8]. Qiming granules, widely studied as an adjunct to artificial tears, include Astragalus (Qi-tonifying) with Rehmannia/Lycium (Yin-nourishing “eyes-brightening”) and Cassia seed, aligning with Qi–Yin dual deficiency [3].

Taken together, these formula families map onto real-world add-on trial designs, particularly most often oral TCM + hyaluronic-acid/artificial tears versus oral placebo + artificial tears. This allows the incremental effect of TCM to be isolated beyond lubrication alone. Additionally, patients with dry eye disease exhibit elevated tear cytokines, and emerging preclinical and early clinical evidence indicates that acupuncture and traditional Chinese medicine can down-modulate inflammatory signaling, supporting a pathophysiology-aligned role for TCM alongside clinical endpoints [9,10].

Despite these encouraging findings, the current evidence base remains fragmented: trials use diverse formula classes and routes, mix comparators, and often embed TCM as add-ons to artificial tears—yet these architectures are rarely grouped and analyzed in ways that reflect real-world practice. Prior reviews typically pool single formulas or narrow subsets, yielding no unified, decision-ready view across key endpoints. Therefore, the objective of this study is to conduct a systematic review and network meta-analysis of randomized trials to evaluate and rank traditional Chinese medicine, as monotherapy or adjunct to standard care, for prevention and management of dry eye disease, synthesizing primary clinical outcomes and secondary inflammatory biomarkers.

## 2. Materials and Methods

### 2.1. Search Strategy

This systematic review and meta-analysis were registered in PROSPERO (CRD420251072475 National Institute for Health Research, Centre for Reviews and Dissemination, University of York, York, UK). The participant–intervention–comparison–outcome–study design (PICOS) framework is presented in Table 1. A comprehensive search of PubMed (National Center for Biotechnology Information, Bethesda, MD, USA), Embase (Elsevier, Amsterdam, The Netherlands), the Cochrane Library (John Wiley & Sons, Ltd., Chichester, UK), CINAHL (EBSCO Information Services, Ipswich, MA, USA), and CNKI (China National Knowledge Infrastructure, Beijing, China) was conducted from inception through June 2025, using Medical Subject Headings and free-text terms related to traditional Chinese medicine, dry eye, and randomized controlled trials (Table S1). The search was restricted to English- and Chinese-language publications. Additional records were identified by screening existing systematic reviews and the reference lists of included studies. Searches and study selection were performed independently by two reviewers, and disagreements were resolved through consultation with a third reviewer.

Eligibility criteria were tailored to TCM and DED. Randomized controlled trials enrolling adults (≥18 years) with DED were included if they investigated TCM—oral decoctions/patent medicines or topical TCM preparations—used as monotherapy or as adjuncts to standard care (e.g., artificial tears). Studies were required to report at least one primary clinical endpoint. Trials were excluded if they: (i) were non-randomized or non-controlled; (ii) evaluated non-herbal TCM modalities only (e.g., acupuncture alone) without a TCM herbal arm; (iii) used non-DED populations or mixed indications without separable DED data; (iv) incorporated unbalanced co-interventions beyond standard care; or (v) were non-human, in vitro, observational, case reports, reviews, or non-English publications. Title/abstract and full-text screening were performed independently by W.H. and Y.J., with discrepancies resolved by discussion (and escalation to a third reviewer if needed).

### 2.2. Data Extraction and Risk of Bias Assessment

After study selection, two reviewers independently extracted the following information: first author’s name, year of publication, study design (randomized controlled trial: parallel or cross-over), population size, mean baseline age, study duration, participant condition and description of the TCM interventions. For primary outcomes, pre- and post-intervention values, along with their corresponding standard deviations, were collected for TBUT, SIT, and FL. For secondary outcomes, inflammatory biomarkers, including interleukin-6 (IL-6), tumor necrosis factor-α (TNF-α), erythrocyte sedimentation rate (ESR), and immunoglobulin G (IgG), were extracted (tear/serum, as reported). The corresponding authors of the selected articles were contacted when additional information was needed. After extraction, each TCM intervention arm was classified into one of five pattern-based classes: Yin–Kidney (YK), Liver–Spleen Regulating (LSR), Qi–Yin Support (QYS), Yin-Moistening (YM), and Blood-Nourishing/Heat-Clearing (BHC), or as a separate Modern node, defined by the core therapeutic principle and representative herbs (Table S9); the class assignment of each included trial is reported in Table 2.

The Risk of Bias 2 (RoB 2) tool, as described in the Cochrane Handbook for Systematic Reviews of Interventions, was utilized to evaluate the risk of bias for each included study [44]. Judgments of low risk, some concerns, or high risk were made across several domains: randomization process, deviations from intended interventions, missing outcome data, outcome measurement, selection of the reported result, and overall risk of bias. A study was considered to have a high risk of bias if one or more domains were rated as high-risk, or if multiple domains raised concerns that collectively affected the confidence in the findings. Two reviewers independently performed the assessments, with any disagreements resolved through discussion with the co-authors.

### 2.3. Data Synthesis and Analysis

For each outcome, the geometry of available evidence was visualized with a network plot, in which nodes represented intervention strategies (e.g., TCM monotherapy, TCM + hyaluronic-acid (HA) artificial tears, HA alone/standard care, placebo, or other active comparators) and edges denoted direct head-to-head trials. Node size was scaled to the total number of randomized participants, and edge thickness reflected the number of contributing studies. To assess the transitivity assumption, the distribution of three pre-specified effect modifiers (disease severity, administration route, and trial architecture) was compared across the nodes of the network (Table S8).

A random-effects network meta-analysis (NMA) was then undertaken to estimate relative effects across all interventions. Primary outcomes were SIT (mm/5 min), TBUT (seconds), and FL (scale as reported). Where trials used the same measurement scale, effects were summarized as weighted mean differences (WMDs) with 95% credible intervals (CrIs) with change-from-baseline contrasts. For FL, which was measured on heterogeneous grading scales, effects were expressed as standardized mean differences (SMDs); for meta-regression, studies using non-comparable grading scales were excluded, and analyses were restricted to studies using the most commonly applied conventional FL scale; effects were therefore expressed as mean differences (MDs) to facilitate clinical interpretation. To facilitate comparative interpretation, surface under the cumulative ranking curve (SUCRA) values were calculated, with higher SUCRA indicating a greater probability of being among the most effective options for improving DED outcomes.

All analyses were conducted in R (RStudio, version 2024.12.1, Vienna, Austria) under a Bayesian framework using Markov chain Monte Carlo (MCMC) as implemented in the BUGSnet package (version 1.0.4; Department of Community Health Sciences, University of Calgary, Calgary, AB, Canada). Posterior distributions were generated for all parameters, with summaries reported as medians and 95% credible intervals (2.5th–97.5th percentiles). Posterior tail-area probabilities were used to quantify evidence; when *p*-values were reported, they were derived from these tail areas, with *p* < 0.05 considered statistically significant.

Vague priors were used for all model parameters (BUGSnet defaults): normal priors for trial baselines and treatment effects, and a uniform prior for the between-study standard deviation, with data-dependent bounds. Three chains were run with 5000 adaptation and 5000 burn-in iterations, followed by 50,000 iterations per chain. Convergence was assessed using the Gelman–Rubin statistic ($\hat{R}$ < 1.1). Heterogeneity was quantified by the between-study standard deviation (τ). Model fit was assessed using the deviance information criterion (DIC), and inconsistency between direct and indirect evidence was assessed by comparing the consistency and inconsistency models.

### 2.4. Certainty of Evidence Assessment

The certainty (confidence) of the network meta-analysis estimates was assessed using the CINeMA framework (Confidence in Network Meta-Analysis, version 2.0.0; Institute of Social and Preventive Medicine, University of Bern, Bern, Switzerland), which evaluates six domains: within-study bias, reporting bias, indirectness, imprecision, heterogeneity, and incoherence [45,46]. Each comparison for the primary outcomes (SIT, TBUT, and FL) was rated as high, moderate, low, or very low confidence.

Within-study bias was informed by the RoB 2 assessments [44]. Reporting bias was assessed narratively, given the limited number of studies per comparison [45]. Indirectness was judged by comparing the populations, interventions, and outcomes between direct and indirect evidence. Imprecision was assessed by whether the 95% credible interval of each comparison crossed the minimally important difference. Heterogeneity was assessed from the posterior between-study standard deviation (τ). Incoherence was assessed by node-splitting (separating indirect from direct evidence), which compares the direct and indirect estimates of each comparison [47].

## 3. Results

### 3.1. Results of the Literature Search

**Figure 1:**
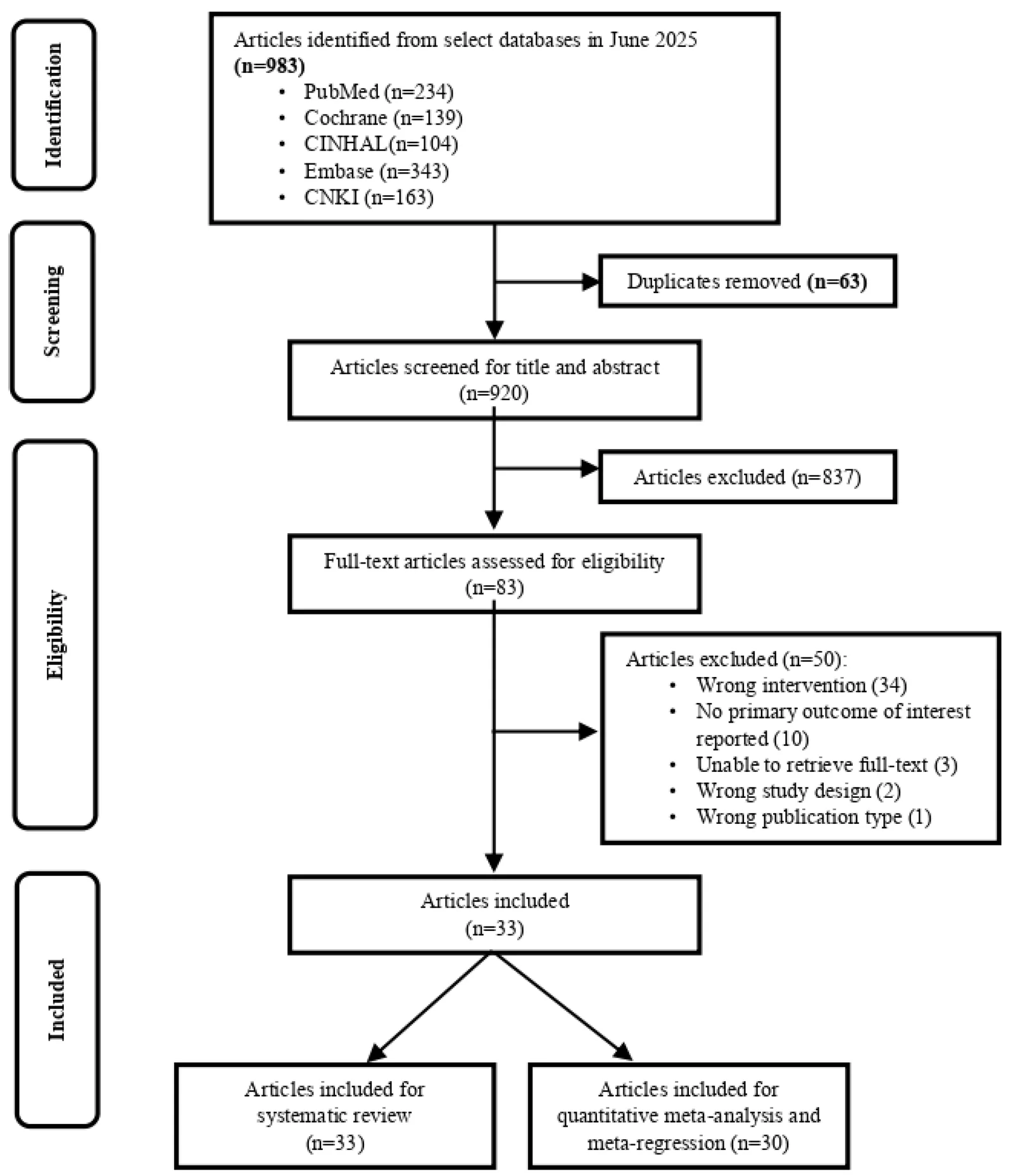
Flow diagram of the literature search process (PRISMA).

As shown in Figure 1, 983 articles were identified from databases in June 2025 (PubMed = 234, Cochrane = 139, CINAHL = 104, Embase = 343, CNKI = 163). After 63 duplicates were removed, 920 articles were screened by title and abstract, of which 837 were excluded as ineligible. The full texts of 83 articles were assessed; 50 were excluded for the following reasons: wrong intervention (*n* = 34), no primary outcome of interest reported (*n* = 10), unable to retrieve full text (*n* = 3), wrong study design (*n* = 2), and wrong publication type (*n* = 1). In total, 33 studies were included in the systematic review, and 30 contributed data to the quantitative network meta-analysis and meta-regression [11,12,13,14,15,16,17,18,19,20,21,22,23,24,25,26,27,28,29,30,31,32,33,34,35,36,37,38,39,40,41,42,43].

### 3.2. Study Characteristics

**Figure 2:**
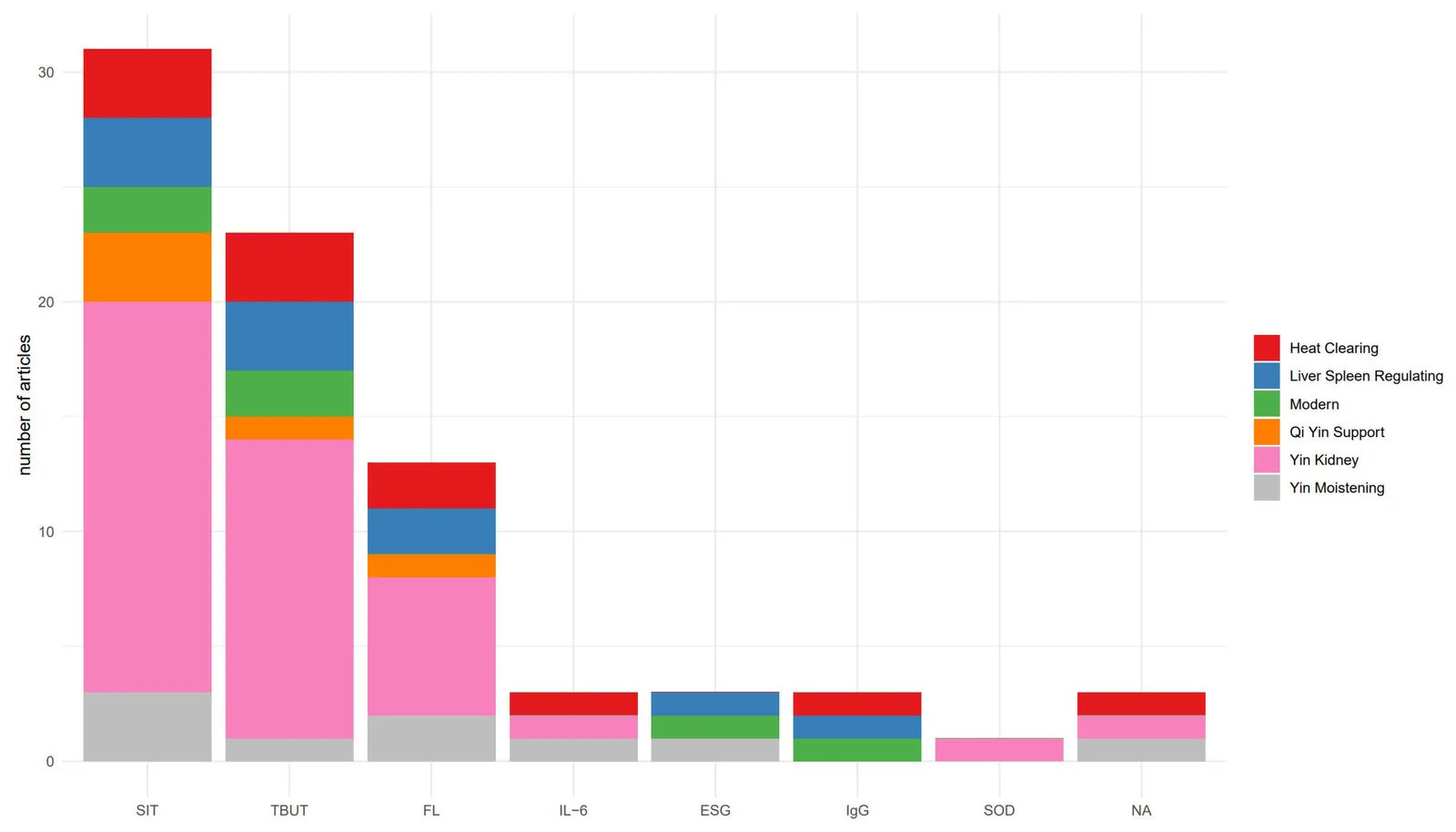
The number of included articles reporting each primary outcome (SIT, TBUT, FL) by TCM class. Evidence is densest for YK, with LSR, QYS, and YM showing comparable numbers of studies on these endpoints, while BHC and Modern are less represented. Reporting of secondary inflammatory outcomes was sparse and heterogeneous across classes (assays, matrices, and timepoints varied). Number of Articles Reporting Primary and Secondary Outcomes. ESG (Erythrocyte Sedimentation Rate); FL (Corneal Fluorescein Staining); IL-6 (Interleukin-6); IgG (Immunoglobulin G); SIT (Schirmer I Test); SOD (Superoxide Dismutase); TBUT (Tear Film Break-Up Time); TNF-α (Tumor Necrosis Factor-alpha).

The study and participant characteristics of the included 33 articles are summarized in Table 2. Most used parallel-group designs with treatment durations clustered at 2–12 weeks (one trial at 24 weeks; a few at 1–3 weeks). Sample sizes per study typically ranged from 21 to 212 participants. Populations included uncomplicated DED (*n* = 17), severe DED (*n* = 9), and post-surgical DED (*n* = 7). As shown in Figure 2, interventions were mapped by route/trial architecture: oral TCM monotherapy, oral TCM + standard care (SC; e.g., hyaluronic-acid/artificial tears), topical TCM monotherapy, topical TCM + SC, and modern/non-TCM extracts. Within TCM arms, formulas were classified by their core therapeutic principles and key components into Yin–Kidney (YK), Liver–Spleen Regulating (LSR), Qi–Yin Support (QYS), Yin-Moistening (YM), or Blood-Nourishing/Heat-Clearing (BHC), based on the dominant principle/ingredients. Most articles evaluated YK (*n* = 17); others included BHC (*n* = 4), LSR (*n* = 4), QYS (*n* = 3), YM (*n* = 3), and Modern (*n* = 2). Comparators reflected practice, with frequent add-on designs (≈20 TCM + SC vs. SC only) and placebo controls (≈13 oral/topical).

### 3.3. Network Diagrams

**Figure 3:**
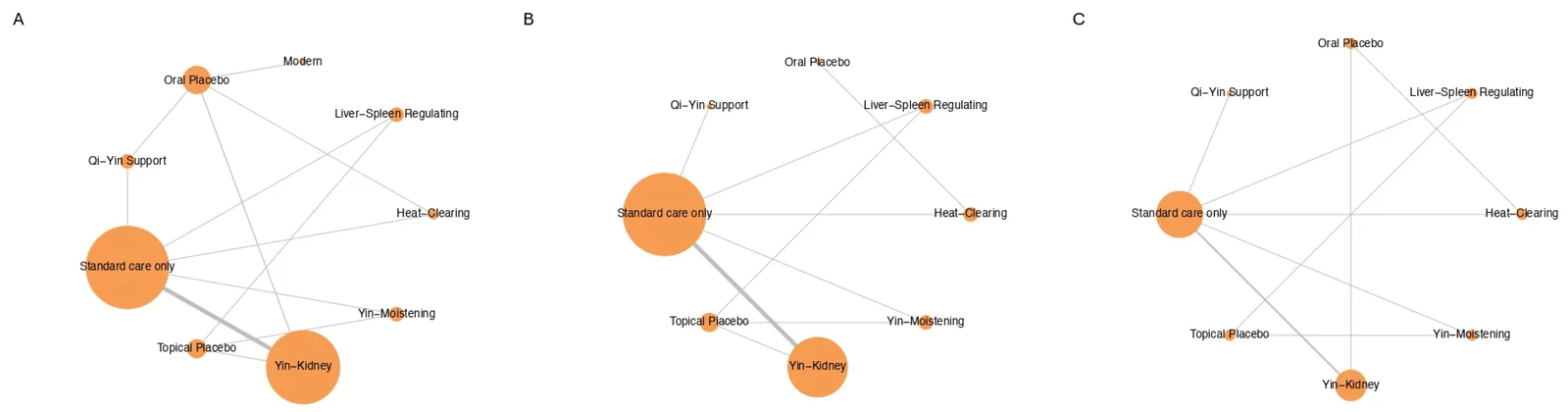
Network diagram for (**A**) SIT, (**B**) TBUT, (**C**) FL. FL (Corneal Fluorescein Staining); SIT (Schirmer I Test); TBUT (Tear Film Break-Up Time). Nodes represent interventions, with node size proportional to the total number of participants included for each intervention. Connecting lines indicate direct comparisons, with line thickness proportional to the number of studies contributing to each comparison.

Network graphs for the three primary outcomes (SIT, TBUT, and FL) based on the above groupings are shown in Figure 3A–C, respectively. Across all outcomes, the most frequent direct comparison was standard care only versus YK, followed by Oral Placebo versus YK; remaining links among LSR, QYS, YM, BHC, and Modern were less common but similarly represented. The number of pairwise comparisons and participant counts for each outcome are provided in Tables S2–S4.

### 3.4. Outcomes

#### 3.4.1. Schirmer I Test (SIT)

**Figure 4:**
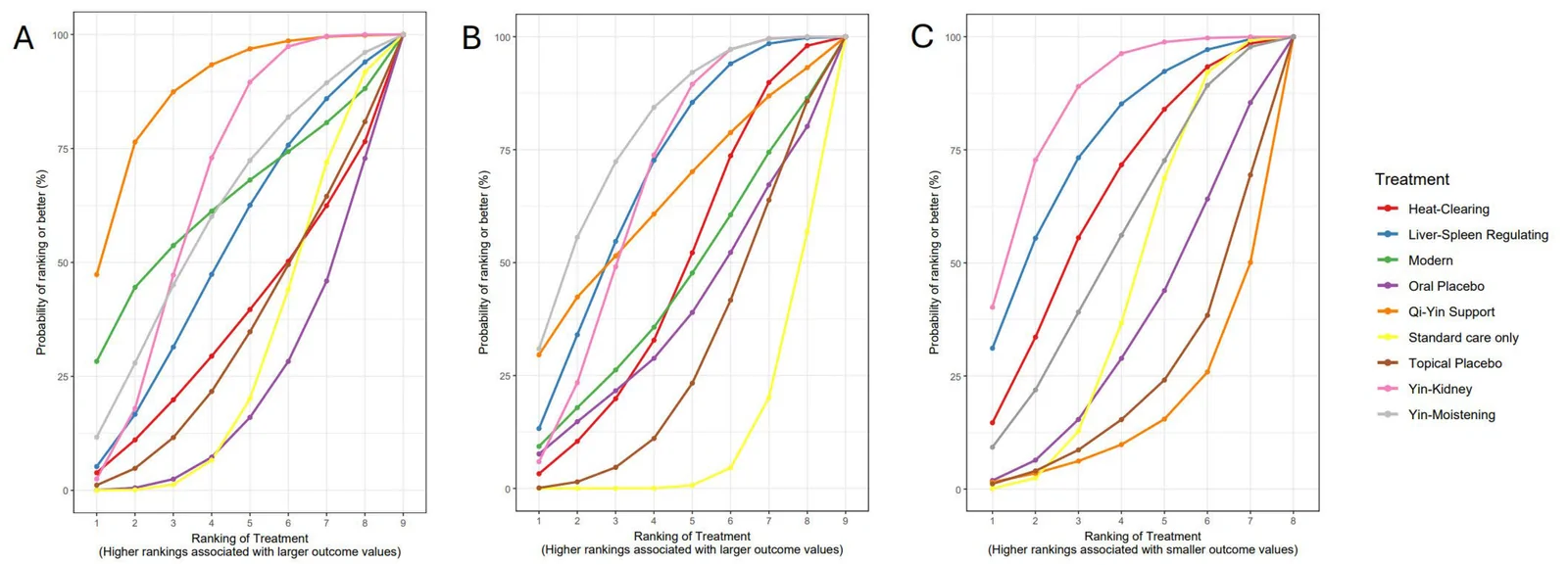
Surface Under the Cumulative Ranking Curve (SUCRA) plot for (**A**) SIT, (**B**) TBUT, (**C**) FL. FL (Corneal Fluorescein Staining); SIT (Schirmer I Test); TBUT (Tear Film Break-Up Time).

Among the nine groups, QYS ranked highest (SUCRA: 87.42%), followed by YK (SUCRA: 65.9%) and Modern (SUCRA: 62.37%); SUCRA rankings for SIT are shown in Figure 4A. Comparator nodes ranked lower: Topical Placebo 33.96%, standard care only 30.41%, and Oral Placebo 22.50%. In pairwise contrasts, QYS outperformed Standard Care (WMD: 5.32, 95% CrI: 0.63 to 10.06) and YK exceeded Standard Care (WMD: 2.56, 95% CrI 0.25 to 4.86); QYS was also superior to Oral Placebo (WMD: 6.18, 95% CrI 0.83 to 11.61). The full league table is provided in Table S5.

#### 3.4.2. Tear Film Break-Up Time (TBUT)

For TBUT, SUCRA rankings (Figure 4B) placed YM highest (79.01%), followed by Liver–Spleen Regulating (SUCRA: 69.04%), YK (SUCRA: 67.31%); the Modern node ranked sixth (44.77%), comparators ranked lower: Oral Placebo, 38.74%; Topical Placebo, 26.80%; and standard care only, 9.27%. As shown in the league table (Table S6), YM, LSR, and YK each showed statistically significant improvements compared with Standard Care: YM (WMD: 2.49, 95% CrI 0.64 to 4.37), LSR (WMD: 2.10, 95% CrI 0.57 to 3.59), YK (WMD: 2.04, 95% CrI 1.12 to 2.89). By contrast, Modern (WMD: 1.24, 95% CrI −1.47 to 3.97) and QYS (WMD: 2.11, 95% CrI −1.10 to 5.33) did not differ significantly from Standard Care. Additionally, YM also demonstrated greater improvement than Topical Placebo (WMD: 1.79, 95% CrI 0.36 to 3.23).

#### 3.4.3. Corneal Fluorescein Staining (FL)

For FL, SUCRA rankings (Figure 4C) placed YK the highest (SUCRA: 85.20%), followed by LSR (SUCRA: 76.52%) and BHC (64.57%), with comparator nodes ranked lower (standard care only 44.59%; Oral Placebo 35.10%; Topical Placebo 23.00%), and QYS ranked lowest (16.19%). In pairwise SMD contrasts versus standard care, YK showed the greatest reduction (SMD −0.37, 95% CrI −0.71 to 0.00), followed by LSR (SMD −0.30, 95% CrI −0.99 to 0.39), whereas QYS showed no improvement (SMD 0.40, 95% CrI −0.39 to 1.20). Exact estimates are provided in Table S7.

#### 3.4.4. Inflammatory Secondary Outcomes

The most frequently assessed markers were IL-6 and TNF-α, with IL-1β, ESR, IgG, and superoxide dismutase (SOD). Across five representative trials, the direction of effect favored TCM for the reported biomarkers and was generally concordant with improvements in the corresponding primary outcomes (SIT, TBUT, FL). By count: IL-6 (n = 2), TNF-α (n = 2), IL-1β (n = 1), ESR (n = 2), IgG (n = 2), SOD (n = 1), MMP-9 (n = 0). Represented classes included YK, QYS, BHC, and YM (Table 3).

#### 3.4.5. Baseline Disease Severity and Changes in DED Outcomes

**Figure 5:**
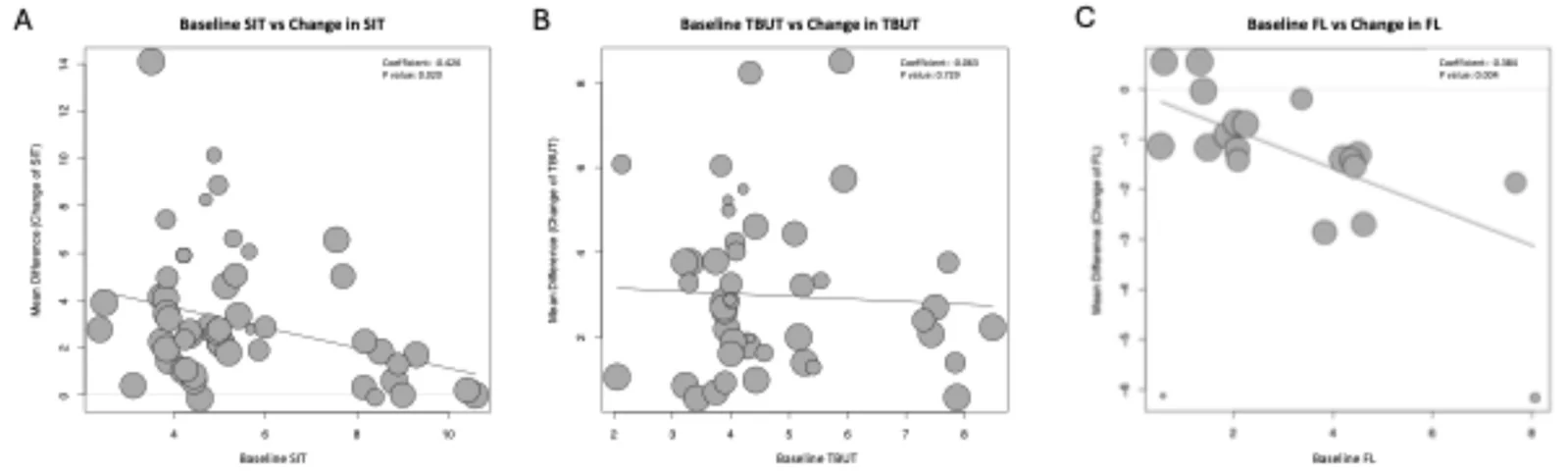
Meta-regression of baseline (**A**) SIT, (**B**) TBUT, (**C**) FL versus change in the respective outcome. FL (Corneal Fluorescein Staining); SIT (Schirmer I Test); TBUT (Tear Film Break-Up Time).

Meta-regression analysis on the relationship between baseline disease severity and changes in SIT, TBUT, and FL shows significant negative associations for SIT (β = −0.426, *p* = 0.020) and FL (β = −0.384, *p* = 0.004), but no significant correlation for TBUT (Figure 5A–C). Further meta-regression by mean age and study duration for each primary outcome did not show significant associations with changes in SIT, TBUT, or FL (Figures S1 and S2, respectively).

#### 3.4.6. Assessment of Transitivity, Risk of Bias, and Sensitivity and Certainty of Evidence

Potential effect modifiers, including study duration, sample size, disease severity, administration route, and trial architecture were examined to assess transitivity. Distributions of age, study duration, male proportion, and sample size were broadly comparable across treatment contrasts, with no imbalances evident (Table S8): add-on designs comprised 60.6% (20/33) of trials, 87.9% (29/33) used oral formulations, and severity subtypes (uncomplicated 51.5%, severe 27.3%, post-surgical 21.2%) were distributed across comparison loops rather than isolated within a single node. Collectively, these findings supported the transitivity assumption and the plausibility of indirect comparisons.

Methodological quality was checked using RoB 2. Twelve trials were rated low-risk overall; twenty-one were rated some concerns, most frequently due to Domain 2 (deviations from intended interventions), where blinding/adherence was insufficiently detailed. No trial was judged high-risk overall (Figure S4). To formally assess the certainty of the NMA estimates, the CINeMA framework was applied, evaluating within-study bias, reporting bias, indirectness, imprecision, heterogeneity, and incoherence for each comparison. Confidence varied across outcomes (Table 4): for SIT, it was moderate (QYS), low (LSR, YM, BHC), and very low (YK); for TBUT, most TCM classes were rated moderate, with QYS and Modern rated low; for FL, for FL, YK, BHC, and YM were rated moderate, whereas LSR and QYS were rated low. Downgrading was driven mainly by within-study bias (Domain 2 concerns), heterogeneity, and imprecision. Accordingly, the NMA estimates should be interpreted with moderate-to-low certainty. In addition to the risk-of-bias assessment, several sensitivity analyses were performed. First, excluding the seven post-surgical trials did not alter the principal rankings: YK remained the top Chinese herbal medicine class for SIT (SUCRA: 74.11%), and Liver–Spleen Regulating remained top for FL (SUCRA: 91.89%). Second, restricting the network to the 18 add-on trials yielded a connected six-node star network in which QYS ranked first (SUCRA: 97.63%) and YK remained robustly superior to standard care (SUCRA 57.62% vs. 20.42%). Lastly, excluding the two modern botanical-extract trials left the SIT hierarchy unchanged (QYS SUCRA: 90.33%; YK SUCRA: 69.05%).

#### 3.4.7. Model Convergence, Heterogeneity, and Inconsistency

Convergence was achieved for all outcomes, with all Gelman–Rubin statistics below 1.1 (maximum: SIT 1.046, TBUT 1.001, FL 1.006). The between-study standard deviation (τ) was largest for SIT (posterior median 3.80, 95% CrI 2.80–5.51), followed by TBUT (1.03, 0.62–1.86) and FL (0.28, 0.04–0.74). Comparing the consistency and inconsistency models showed no substantial incoherence (all ΔDIC < 5): SIT 107.46 vs. 108.15; TBUT 81.57 vs. 83.32; FL 51.50 vs. 49.54 (ΔDIC = 1.96).

## 4. Discussion

This first-of-its-kind network meta-analysis of 33 randomized trials classified TCM interventions by pattern and trial architecture and ranked their comparative effectiveness, directly addressing fragmented evidence and the common mixing of monotherapy with add-on designs. Consistent improvements were observed with TCM, and differential responses across TCM groups were seen for specific DED outcomes, suggesting that distinct clinical presentations may benefit from different TCM strategies. Several classes outperformed standard care in add-on settings. Secondary inflammatory biomarkers generally trended concordantly with clinical benefit, and meta-regression indicated that treatment effects varied with baseline severity, pointing to baseline severity as a modifier of effect.

Across included RCTs, SIT, TBUT, and FL were most frequently reported; evidence was densest for YK, with smaller bodies for LSR, YM, BHC, QYS, and Modern. Improvements across SIT/TBUT/FL likely reflect (1) conceptual fit—many tested formulas align with TCM’s Qi–Yin deficiency framework for DED (e.g., QYS, YK), targeting tear/fluid replenishment [48]; (2) clinical relevance—these classes map to the common aqueous-deficient presentation, where gains in SIT and downstream stability (TBUT) are plausible [49]; and (3) feasibility/standardization—widely available, well-tolerated, and relatively standardized patent granules/capsules support consistent dosing, adherence, and multi-site implementation, enabling clearer signal detection in trials. Sparsely reported secondary outcomes may be a result of assay/logistical constraints and a historical emphasis on clinical efficacy over mechanistic validation; this limits mechanistic inference, underscoring the need for future trials to co-prioritize clinical and mechanistic endpoints with harmonized assays/time points.

To contextualize comparative estimates, network plots were constructed to visualize the geometry of evidence across interventions and comparators. Standard care only (typically HA/artificial tears) and YK formed the backbone of the network. Standard care was the most common comparator because lubricants are first-line management for DED, representing the clinical baseline and allowing the incremental benefit of adding TCM to be assessed. Evidence syntheses and TFOS DEWS II guidance support artificial tears—particularly HA formulations—as a core therapy with measurable effects on primary outcomes, justifying their role as the anchor node [50,51]. Oral placebo was frequently used for oral TCM trials because it matches the route of administration and controls expectancy effects, which is the methodologically preferred approach for minimizing bias and preserving assay sensitivity in randomized trials.

In the NMA, QYS showed a differential profile across outcomes, ranking first for SIT, second for TBUT, but last among TCM groups for FL reduction. This profile is consistent with its TCM methodology: QYS formulas such as Qiming are designed to nourish qi and yin fluids, thereby supporting lacrimal gland function and directly boosting aqueous tear secretion, which plausibly underlies the top ranking for SIT—a standard clinical measure of basal and reflex aqueous tear production [3,52]. Moreover, when yin is replenished and surface tissues are better hydrated, the aqueous layer becomes thicker, the epithelium less rough and damaged, and mucin distribution more uniform across the ocular surface; all of which can delay tear film break-up and improve tear film adherence and stability, helping to explain the strong (though not dominant) performance of QYS in TBUT [53,54]. However, QYS primarily addresses “yin-deficiency-induced dryness” rather than “blood stasis” or “heat/toxin accumulation,” which are more closely linked to epithelial damage and staining; as a result, it is less effective at repairing ocular-surface defects, consistent with its weaker impact on FL [55,56]. In contrast, other TCM groups such as BHC include formulas that more directly target inflammation, microcirculatory disturbance, and tissue repair, and therefore tend to outperform QYS in FL improvement.

By contrast, YK formulas, represented clinically by Qi Ju Di Huang Wan and broader Lycium–Rehmannia pill combinations, show a more balanced, multi-domain profile across outcomes. A randomized trial of Chi-Ju-Di-Huang-Wan demonstrated improvements in tear film stability and reduced corneal epithelial abnormality in DED patients, while a meta-analysis of Lycium–Rehmannia pills plus conventional therapy showed gains in SIT, TBUT, and FL, along with symptom relief [7,11]. These findings are consistent with the YK rationale of “nourishing Liver–Kidney yin” to support tear secretion, tear film stability, and epithelial repair, making YK suitable for multifactorial DED with combined secretion, stability, and surface-damage deficits.

LSR formulas which ranked second for FL reduction can be interpreted considering both nutrient-support and anti-inflammatory mechanisms. Representative LSR prescriptions such as Chaihu Shugan San/Chaihu Shugan powder have been shown to improve gut microbiota and enhance digestive function in dyspepsia models, which would be expected to support systemic nutrient supply for epithelial repair, including at the ocular surface [57]. In parallel, multiple preclinical studies in liver and metabolic disease models demonstrate that Chaihu Shugan-type formulas down-regulate TNF-α and IL-6 which are cytokines that are also upregulated on the ocular surface in DED [58,59].

Meta-regression showed that lower baseline SIT was associated with larger SIT improvements, and higher baseline FL with greater FL reductions, whereas baseline TBUT was not significantly related to change in TBUT. This pattern is consistent with DED pathophysiology: reduced SIT primarily reflects aqueous-deficient DED, and higher fluorescein staining reflects more extensive ocular surface epithelial damage and inflammation, so more severe baseline “deficits” in secretion or epithelial integrity naturally allow greater absolute gains when those domains are effectively targeted [1,55,60]. In contrast, TBUT is a composite endpoint shaped by aqueous volume, mucin and lipid layers, and blink/lid dynamics, and clinical data suggest that it often shows only modest and variable correlations with other DED metrics such as osmolarity and symptoms rather than a simple linear deficit–response pattern [1,61]. Nevertheless, these meta-regression findings should be interpreted with caution, as regressing change scores against baseline values in study-level aggregate meta-analyses is susceptible to regression-to-the-mean effects and ecological bias.

The strengths of this NMA include a prespecified TCM classification framework, explicit separation of monotherapy vs. add-on designs, use of network meta-analysis with meta-regression, and parallel consideration of clinical and mechanistic outcomes, providing a structured, mechanism-aware comparison of TCM groups. However, most trials were conducted in Asian settings, with generally small sample sizes, variable risk of bias and limited biomarker reporting, so publication and regional bias cannot be excluded and generalizability to more diverse, non-Asian populations remain uncertain. In addition, the add-on-only sensitivity network contained no closed loops, precluding formal inconsistency assessment, and the ranking of QYS was sensitive to the inclusion of its two studies, warranting cautious interpretation.

## 5. Conclusions

This network meta-analysis classified TCM interventions for DED into pattern-based groups and ranked their comparative effectiveness across SIT, TBUT, and FL. Effects varied by outcome: YM and LSR showed the largest gains in TBUT, YK and LSR were most effective for reducing FL, and YK and QYS yielded the greatest improvements in SIT. In head-to-head comparisons, YK/QYS showed statistically significant improvements in both tear production and stability (SIT and TBUT), whereas LSR led for ocular-surface repair (FL). Meta-regression suggested larger benefits in eyes with greater baseline tear-production deficits, and more severe corneal staining, indicating that patients with more compromised baseline tear secretion and ocular-surface integrity may derive greater absolute improvements from treatment. Overall, the findings support a pathophysiology-aligned role for TCM as an adjunct to lubricants in DED and provide a comparative framework to guide formula selection by clinical pattern and outcome priority.

## Figures and Tables

**Table 1:** PICOS.

Criteria	Description
Participants	Adults (≥18 years), including both: healthy volunteers (in studies assessing preventive or subclinical effects) patients diagnosed with dry eye disease by established criteria (from TFOS DEWS II)
Interventions	Any Chinese herbal medicine; topical (e.g., eye drops, ointments) or systemic (oral decoctions, granules); administered alone or adjunctively, with defined dosing and duration.
Comparators	Different treatment as the intervention group including placebo (ex. saline eye drops) OR artificial tears OR conventional pharmacotherapy (ex. cyclosporine, hyaluronic acid) OR no treatment/wait-list control
Outcomes	Primary: Tear-film break-up time (TBUT); Schirmer I test (SIT); Corneal fluorescein staining score (fluorescein grading scales; FL). Secondary: Inflammatory biomarkers and pathway biomarkers linked to DED pathophysiology
Setting	Randomized Controlled Trial

**Table 2:** Characteristics of the randomized controlled trials included in the systematic review and network meta-analysis.

Study ID	Author (Year)	Study Duration (Weeks)	Description Intervention/Placebo	Population Size	Condition	Conclusion
1	Chang, Lin (2005) [11]	4	Yin–Kidney	40	Dry eye	Chi-Ju-Di-Huang-Wan improved tear secretion function, promoted tear film stabilization and promoted corneal damage repair.
			Standard care only	40		
2	Chen, Chen (2014) [12]	4	Yin–Kidney	31	Dry eye	Yangyin Shengjin and Buyi Ganshen improved tear secretion function, promoted tear film stabilization and promoted corneal damage repair.
			Standard care only	31		
3	Chen, Chen (2015) [13]	4	Yin–Kidney	38	Dry eye after surgery	Modified Chi-Ju-Di-Huang-Wan Decoction improved tear secretion function and promoted tear film stabilization.
			Standard care only	38		
4	Chen, Wang (2006) [14]	12	Qi–Yin Support	30	Dry eye after surgery	Modified Adenophora and Ophiopogon Decoction had a promoting effect on TBUT, SIT, and decreased FL in the treatment of dry eye after photorefractive keratectomy.
			Standard care only	30		
5	Fan, Hung (2024) [15]	24	Yin–Kidney	30	Severe dry eye	Cassia tea possesses pronounced antioxidant activity and improved tear secretion function.
			Oral Placebo	30		
6	Gao, Chen (2022) [16]	4	Yin–Kidney	69	Dry eye after surgery	The combination of sodium vitrate eye drops and oral Yin-Nourishing and Eye-Brightening Decoction improved tear film stability, promoted corneal damage repair, and reduced ocular surface inflammation.
			Standard care only	69		
7	Han, Dong (2023) [17]	12	Qi–Yin Support	30	Severe dry eye	The combination of Modified Shengmai Formula improved the function of tear secretion.
			Oral Placebo	30		
8	Heng (2020) [18]	4	Yin–Kidney	60	Severe dry eye	Yangyin Run Eye Soup combined with polyethylene glycol eyedrops improved tear secretion function, promoted tear film stabilization and promoted corneal damage repair.
			Standard care only	60		
9	Hu, Qin (2018) [19]	4	Heat-Clearing	81	Dry eye after surgery	Combined use of ChuFeng YiSunTang and polyethylene glycol eye drops improved tear secretion function and promoted tear film stabilization and promotes corneal damage repair.
			Standard care only	81		
10	Hui, Ni (2022) [20]	2	Liver–Spleen Regulating	30	Severe dry eye	Modified Danzhi Xiaoyao Powder improved tear secretion function, promoted tear film stabilization and promoted corneal damage repair.
			Topical Placebo	30		
11	Kan, Wang (2020) [21]	12	Modern	25	Dry eye	Both 10-mg and 14- mg lutein formula groups had improved tear secretion and tear break-up time.
			Oral Placebo	25		
12	Leng (2018) [22]	3	Yin–Kidney	50	Dry eye	The combination of Qi Ju Di Huang Wan and PEG eye drops for local treatment improved tear secretion function, promoted tear film stabilization and promoted corneal damage repair.
			Standard care only	50		
13	Lin, Pang (2020) [23]	1	Heat-Clearing	30	Dry eye	Coptis Root promoted tear film stabilization.
			Standard care only	30		
14	Liu, Jin (2020) [24]	4	Heat-Clearing	118	Dry eye	Houttuynia Eye Drops Atomization Treatment improved tear secretion function and promoted tear film stabilization.
			Oral Placebo	120		
15	Long (2014) [25]	12	Yin–Kidney	80	Dry eye	Jingqi Mingmu Decoction improved tear secretion function and promoted tear film stabilization.
			Standard care only	80		
16	Nagaki (2003) [26]	12	Yin–Kidney	25	Dry eye	Goshajinkigan improved tear secretion function and promoted corneal damage repair.
			Oral Placebo	25		
17	Ou, Wang (2025) [27]	12	Heat-Clearing	45	Dry eye	Jiedu Tongluo Shengjin promoted tear film stabilization.
			Oral Placebo	44		
18	Pu (2010) [28]	4	Yin–Kidney	60	Dry eye	Xuanmai Dihuang Decoction improved tear secretion function and promoted tear film stabilization.
			Oral Placebo	60		
19	Rolando (2007) [29]	12	Modern	21	Dry eye	TSP 0.5% and 1% demonstrated superior tear-film-stabilizing effects compared with 0.2% HA.
			Standard care only	9		
20	Song (2013) [30]	4	Liver–Spleen Regulating	34	Severe dry eye	Liver-Soothing and Spleen-Strengthening Formula improved tear secretion function and promoted tear film stabilization.
			Oral Placebo	29		
21	Tao, Chen (2016) [31]	4	Yin-Moistening	60	Dry eye	Yin-Nourishing Lung-Clearing Decoction Combined with Shengmai Powder improved tear secretion function, promoted tear film stabilization and promoted corneal damage repair.
			Standard care only	60		
22	Wang, Li (2011) [32]	4	Yin-Moistening	30	Dry eye	Yin-Nourishing Lung-Clearing Decoction improved tear secretion function, promoted tear film stabilization and promoted corneal damage repair.
			Topical Placebo	30		
				96		
23	Xia, Yang (2018) [33]	3	Yin–Kidney	54	Dry eye	Traditional Chinese Medicine Decoction for Nourishing Liver and Kidney, Relieving Muscles and Dredging Collaterals improved tear secretion function and promoted tear film stabilization.
			Topical Placebo	52		
24	Xiang, Zhang (2011) [34]	12	Yin–Kidney	36	Dry eye	Essence-Nourishing, Wolfberry-Eye-Brightening Decoction improved tear secretion function and promoted tear film stabilization.
			Standard care only	36		
25	Xie, Luo (2018) [35]	4	Liver–Spleen Regulating	30	Dry eye	Sini powder improved tear secretion function and promoted tear film stabilization.
			Standard care only	30		
26	Xu, Ye (2010) [36]	4	Yin–Kidney	24	Severe dry eye	The treatment group significantly increased tear secretion in dry-eye patients and was superior to the control group. Both the treatment and control groups markedly improved tear-film stability, with no significant between-group difference after treatment.
			Standard care only	24		
27	Yang, Zhang (2014) [37]	8	Yin–Kidney	148	Severe dry eye	Xuanmai Eye-Moistening Decoction improved tear secretion function and promoted tear film stabilization.
			Standard care only	128		
28	Yi, Zhang (2018) [38]	4	Yin–Kidney	36	Dry eye	Mingmu Dihuang Formula Combined with Shengmai Powder improved tear secretion function and promoted tear film stabilization
			Standard care only	36		
29	Yu, Zeng (2012) [39]	4	Yin–Kidney	120	Severe dry eye	Chi-Ju-Di-Huang-Wan improved tear secretion function, promoted tear film stabilization and promoted corneal damage repair.
			Standard care only	120		
30	Zeng, Xia (2014) [40]	2	Liver–Spleen Regulating	114	Severe dry eye after surgery	Traditional Chinese Medicine Granules for Clearing Liver and Relieving Depression, Nourishing Blood and Activating Blood Circulation, Nourishing Yin and Moistening Eyes improved tear secretion function, promoted tear film stabilization and promoted corneal damage repair.
			Standard care only	98		
31	Zeng, Xia (2014) [41]	2	Yin–Kidney	212	Severe dry eye after surgery	TCM improved tear secretion function, promoted tear film stabilization and promoted corneal damage repair.
			Standard care only	202		
32	Zhang, Wang (2015) [42]	4	Qi–Yin Support	32	Severe dry eye	Ganlu Decoction improved tear secretion function, promoted tear film stabilization and promoted corneal damage repair.
			Standard care only	32		
33	Zhao (2017) [43]	4	Yin-Moistening	62	Dry eye after surgery	Traditional Chinese Medicine for Promoting Fluid Production and Moistening Dryness, Clearing and Dispersing Lung Qi improved tear secretion function, promoted tear film stabilization and inhibited inflammation.
			Topical Placebo	62		

**Table 3:** Secondary inflammatory outcomes.

Author (Year)	Arms	Reported Secondary Biomarker	Matrix & Assay	Study Duration (Weeks)	Direction of Effect	Consistency with Clinical Outcome
Fan, Hung (2025) [15]	YK vs. Oral Placebo	SOD	Serum	8	SOD favors TCM	SIT favors TCM
Gao, Chen (2022) [16]	YK vs. Standard Care	TNF-α, IL-6, IL-1β	Tear sample	4	All favors TCM	SIT, TBUT and FL favors TCM
Han, Dong (2023) [17]	QYS vs. Oral Placebo	ESR, IgG	Serum	12	All favors TCM	SIT favors TCM
Ou, Wang (2025) [27]	BHC vs. Standard care only	ESR, IgG	Serum	12	All favors TCM	TBUT favors TCM
Zhao (2017) [43]	YM vs. Topical Placebo	TNF-α, IL-6	Tear sample	4	All favors TCM	SIT and TBUT favors TCM

**Table 4:** CINeMA confidence ratings for each treatment class versus standard care.

Outcome	YK	LSR	QYS	YM	BHC	Modern
SIT	Very low	Low	Moderate	Low	Low	Very low
TBUT	Moderate	Moderate	Low	Moderate	Moderate	Low
FL	Moderate	Low	Low	Moderate	Moderate	—

## Data Availability

The original contributions presented in this study are included in the article/Supplementary Material. Further inquiries can be directed to the corresponding authors.

## Supplementary Materials

The following supporting information can be downloaded at https://www.mdpi.com/article/10.3390/vision10040076/s1: Table S1: Search Strings and Limits for Each Database; Table S2: Details for Network Diagram (SIT); Table S3: Details for Network Diagram (TBUT); Table S4: Details for Network Diagram (FL); Table S5: SIT League Table; Table S6: TBUT League Table; Table S7: FL League Table; Table S8: Clinical characteristics and methodological features of included studies; Table S9: Classification framework for traditional Chinese medicine interventions. Figure S1: Meta-regression of mean age versus change in (A) SIT, (B) TBUT, and (C) FL, respectively; Figure S2: Meta-regression of duration (weeks) versus change in (A) SIT, (B) TBUT, and (C) FL, respectively; Figure S3: Heterogeneity assessment with respect to potential effect modifiers: (A) age, (B) duration (weeks), (C) gender (% male), and (D) sample size; Figure S4: Risk of bias proportion graph (as percentage) for all included randomized controlled trials.

## Abbreviations

The following abbreviations are used in this manuscript:

BHC	Blood-Nourishing/Heat-Clearing
CrIs	Credible intervals
DED	Dry eye disease
ESR	Erythrocyte sedimentation rate
FL	Fluorescein staining
HA	Hyaluronic-acid
IgG	Immunoglobulin G
IL-6	Terleukin-6
LSR	Liver–Spleen Regulating
MCMC	Markov chain Monte Carlo
NMA	Random-effects network meta-analysis
QYS	Qi–Yin Support
RoB 2	Risk of Bias 2
SMD	Standardized mean difference
SIT	Schirmer I test
SUCRA	Cumulative ranking curve
TBUT	Tear film break-up time
TCM	Traditional Chinese medicine
TNF-α	Tumor necrosis factor-α
WMDs	Weighted mean differences
YK	Yin–Kidney
YM	Yin-Moistening

## References

[1] Craig J.P., Nichols K.K., Akpek E.K., Caffery B., Dua H.S., Joo C.-K., Liu Z., Nelson J.D., Nichols J.J., Tsubota K. (2017). TFOS DEWS II Definition and Classification Report. Ocul. Surf..

[2] Perry H.D. (2008). Dry eye disease: Pathophysiology, classification, and diagnosis. Am. J. Manag. Care.

[3] Yang M., Hu Z., Yue R., Yang L., Zhang B., Chen Y. (2020). The Efficacy and Safety of Qiming Granule for Dry Eye Disease: A Systematic Review and Meta-Analysis. Front. Pharmacol..

[4] Tong L., Htoon H.M., Hou A., Acharya R.U., Tan J.-H., Wei Q.-P., Lim P. (2018). Acupuncture and herbal formulation compared with artificial tears alone: Evaluation of dry eye symptoms and associated tests in randomised clinical trial. BMJ Open Ophthalmol..

[5] Yu J., Guo Y., Tian L., Qiu L., Chen X., Cao K., Zhu L.B., Jie Y. (2024). Investigation of traditional Chinese medicine constitution in individuals afflicted by dry eye disease: A retrospective study. Medicine.

[6] Tong K., Lian Q., Tang W., Chen X., Chen F., Wang Z., Qiao S., Han H., Yang C. (2025). Systematic analysis of outcome indicators in traditional Chinese medicine clinical research for dry eye: A review. Medicine.

[7] Lou L., Fu H., Liu H. (2020). The clinical efficiency of lycium-rehmannia pills in treating dry eye symptom: A meta-analysis. Medicine.

[8] Dvm S.E.B. (2021). Qi Ju Di Huang Wan (Lycium Fruit, Chrysanthemum and Rehmannia Pill). Am. J. Tradit. Chin. Vet. Med..

[9] Roda M., Corazza I., Reggiani M.L.B., Pellegrini M., Taroni L., Giannaccare G., Versura P. (2020). Dry Eye Disease and Tear Cytokine Levels—A Meta-Analysis. Int. J. Mol.Sci..

[10] Hu Z., Chen X., Hu Q., Zou M., Liu Z. (2025). Role of Chinese Medicine Monomers in Dry Eye Disease: Breaking the Vicious Cycle of Inflammation. Pharmacol. Res. Perspect..

[11] Chang Y.-H., Lin H.-J., Li W.-C. (2005). Clinical evaluation of the traditional chinese prescription Chi-Ju-Di-Huang-Wan for Dry Eye. Phytother. Res..

[12] Chen C.H., Chen G.X. (2014). Clinical observation of Chinese herbal medicine combined with artificial tears for dry eye with the liver-kidney yin-deficiency pattern. J. Zhejiang Chin. Med. Univ..

[13] Chen D.Q., Chen G.X., Xu D. (2015). Clinical observation of Chinese herbal medicine combined with artificial tears for dry eye after cataract surgery. J. Yunnan Coll. Tradit. Chin. Med..

[14] Chen Y.B., Wang W., Mai C.Y., Wang J., Gan H.X. (2006). Clinical observation of modified Shashen Maidong Decoction for dry eye after laser-assisted in situ keratomileusis. J. Jiangxi Coll. Tradit. Chin. Med..

[15] Fan M., Hung J.-L., Hung S.-H., Chen L.-C., Horng C.-T. (2024). Improvement of Presbyopia, Dry Eye, Intraocular Pressure, and Near Vision Through Cassiae Tea Consumption. Medicina.

[16] Gao L., Chen K.F., Jiang C., Bao X.Y. (2022). Clinical observation of oral Ziyin Mingmu Decoction combined with sodium hyaluronate eye drops for dry eye after laser-assisted in situ keratomileusis. J. Sichuan Tradit. Chin. Med..

[17] Han T.T., Dong W., Xue J.L., Guo L.L., Jia F., Suo X.Q., Yan R. (2023). Clinical observation of Shengmai Yin combined with hydroxychloroquine for Sjogren’s syndrome. Guangming J. Chin. Med..

[18] Heng K.S. (2020). Clinical observation of Yangyin Runmu Decoction for dry eye with the liver-kidney yin-deficiency pattern. Guangming J. Chin. Med..

[19] Hu Y., Qin H., Su L.Y., Pan H.L., Zhang M.M. (2018). Clinical observation of Chufeng Yisun Decoction combined with polyethylene glycol eye drops for dry eye after cataract surgery. China J. Chin. Ophthalmol..

[20] Hui S.Y., Ni S. (2022). Clinical observation of modified Danzhi Xiaoyao Powder combined with artificial tears for dry eye with the liver-meridian stagnation-heat pattern. Chin. Arch. Tradit. Chin. Med..

[21] Kan J., Wang M., Liu Y., Liu H., Chen L., Zhang X., Huang C., Liu B.Y., Gu Z., Du J. (2020). A novel botanical formula improves eye fatigue and dry eye: A randomized, double-blind, placebo-controlled study. Am. J. Clin. Nutr..

[22] Leng Z.Y. (2018). Clinical observation of Qiju Dihuang Pills combined with polyethylene glycol eye drops for dry eye in patients with type 2 diabetes mellitus. Diabetes New World.

[23] Lin W.E., Pang L. (2020). Clinical observation of nebulized Huanglian eye drops combined with sodium hyaluronate eye drops for dry eye with the damp-heat pattern. Chin. J. Ethnomedicine Ethnopharmacy.

[24] Liu Z., Jin M., Li Y., Liu J., Xiao X., Bi H., Pan Z., Shi H., Xie X., Zhang M. (2020). Efficacy and Safety of Houttuynia Eye Drops Atomization Treatment for Meibomian Gland Dysfunction-Related Dry Eye Disease: A Randomized, Double-Blinded, Placebo-Controlled Clinical Trial. J. Clin. Med..

[25] Long F.Z. (2014). Clinical observation of Jingqi Mingmu Decoction for conjunctivochalasis with the liver-kidney yin-deficiency pattern. Asia-Pac. Tradit. Med..

[26] Nagaki Y., Hayasaka S., Hayasaka Y., Kadoi C., Sekiya N., Terasawa K., Sakakibara I. (2003). Effects of Goshajinkigan on Corneal Sensitivity, Superficial Punctate Keratopathy and Tear Secretion in Patients with Insulin-dependent Diabetes Mellitus. Am. J. Chin. Med..

[27] Hospital S.B.D., Ou Y.-S., Wang Y.-L., Zheng P.-P., Xue L., Gao F.-Y., Shanghai University of Traditional Chinese Medicine (2025). Effect of the Jiedu Tongluo Shengjin formulation on plasma immunoglobulin G (IgG) levels in patients with primary Sjögren’s syndrome. Bol. Latinoam. Caribe Plantas Med. Aromáticas.

[28] Pu H.S. (2010). Clinical observation of Xuanmai Dihuang Decoction in 30 patients with dry eye with the lung-kidney yin-deficiency pattern. Guid. J. Tradit. Chin. Med. Pharm..

[29] Rolando M., Valente C. (2007). Establishing the tolerability and performance of tamarind seed polysaccharide (TSP) in treating dry eye syndrome: Results of a clinical study. BMC Ophthalmol..

[30] Song Y. (2013). Clinical observation of Shugan Jianpi Formula for dry eye associated with meibomian gland duct obstruction. Guangming J. Chin. Med..

[31] Tao R.S., Chen M.P., Jing W.T. (2016). Clinical observation of Chinese herbal medicine combined with artificial tears for aqueous-deficient dry eye. China Mod. Dr..

[32] Wang W., Li Y., Feng X.M., Li Q.Y. (2011). Clinical observation of Yangyin Qingfei Decoction for dry eye with the lung-yin deficiency pattern. New Chin. Med..

[33] Xia H., Yang X. (2018). Clinical observation of a method for nourishing the liver and kidney, releasing the muscles, and unblocking the collaterals in the treatment of visual display terminal-associated asthenopia. Pharmacol. Clin. Chin. Mater. Medica.

[34] Xiang M.H., Zhang X.R., Li Q.S., Liu L.M., Zhou H.M., Han Z.M. (2011). Clinical observation of Jingqi Mingmu Decoction for conjunctivochalasis with the liver-kidney yin-deficiency pattern. China J. Chin. Ophthalmol..

[35] Xie Z., Luo J.P., Yan X.W., Zhang L., Zhang X.T., Yuan J.Y., Ze Y. (2018). Clinical observation of Sini Powder for dry eye with the liver-spleen disharmony pattern. Guangming J. Chin. Med..

[36] Xu J.J., Ye H.J. (2010). Clinical observation of Shihu Yeguang Pills combined with hypromellose 2910, dextran 70, and glycerol eye drops for dry eye with the liver-kidney yin-deficiency pattern. J. Chengdu Univ. Tradit. Chin. Med..

[37] Yang Y.Q., Zhang Y., Zhou J. (2014). Clinical observation of Xuanmai Runmu Decoction for dry eye. J. Sichuan Tradit. Chin. Med..

[38] Yi L.B., Zhang Y.L., Yu N.N. (2016). Clinical observation of Mingmu Dihuang Formula combined with Shengmai Powder for dry eye with the liver-kidney yin-deficiency pattern. Hebei J. Tradit. Chin. Med..

[39] Yu Y.J., Zeng Z.C. (2012). Clinical observation of Qiju Dihuang Pills combined with topical artificial tears for dry eye with the liver-kidney yin-deficiency pattern. Inn. Mong. J. Tradit. Chin. Med..

[40] Zeng Z.M., Xia H.Y., Li Y., Song C.Y. (2014). Clinical observation of integrated Chinese and Western medicine for post-LASIK dry eye with the liver depression transforming into fire pattern. New Chin. Med..

[41] Zeng Z.M., Xia H.Y., Li Y., Song C.Y. (2014). Clinical observation of Chinese herbal medicine combined with artificial tears for dry eye after LASIK. China J. Chin. Ophthalmol..

[42] Zhang F.M., Wang J.J., Zhang Q.L. (2015). Clinical observation of Ganlu Yin for dry eye with the damp-heat injuring-yin pattern. China J. Chin. Ophthalmol..

[43] Zhao R.L. (2017). Clinical observation of oral Chinese herbal medicine combined with Dendrobium officinale eye drops for dry eye after cataract surgery. China J. Chin. Ophthalmol..

[44] Sterne J.A.C., Savović J., Page M.J., Elbers R.G., Blencowe N.S., Boutron I., Cates C.J., Cheng H.-Y., Corbett M.S., Eldridge S.M. (2019). RoB 2: A revised tool for assessing risk of bias in randomised trials. Br. Med. J..

[45] Nikolakopoulou A., Higgins J.P.T., Papakonstantinou T., Chaimani A., Del Giovane C., Egger M., Salanti G. (2020). CINeMA: An approach for assessing confidence in the results of a network meta-analysis. PLoS Med..

[46] CINeMA: Software for Confidence in Network Meta-Analysis. Institute of Social and Preventive Medicine, University of Bern. https://cinema.ispm.unibe.ch.

[47] Dias S., Welton N.J., Caldwell D.M., Ades A.E. (2010). Checking consistency in mixed treatment comparison meta-analysi. Stat. Med..

[48] Xie M., Cai Z., Li J., Tan J., Liu P., Yu Y., Jiao L. (2024). Metabolic characteristics of Qi-Yin deficiency and heat stagnation in liver meridian patterns of dry eye based on tear metabolomics. Digit. Chin. Med..

[49] Sabucedo-Villamarin B., Garcia-Queiruga J., Cacabelos-Torres L., Giraldez M.J., Yebra-Pimentel E., Pena-Verdeal H. (2025). Short-Term Changes in Tear Film Stability and Tear Volume Following the Application of Various DED Management Options in a Healthy Young Population. J. Pers. Med..

[50] Wolffsohn J.S., Semp D.A., Dutta D., Jones L., Craig J.P. (2025). Clinical practice patterns in the management of dry eye disease: A TFOS international survey 2023-24. Ocul. Surf..

[51] Semp D.A., Beeson D., Sheppard A.L., Dutta D., Wolffsohn J.S. (2023). Artificial Tears: A Systematic Review. Clin. Optom..

[52] Su S.-H., Ho T.-J., Yang C.-C. (2021). Retrospective evaluation of the curative effect of traditional Chinese medicine on dry eye disease. Tzu Chi Med. J..

[53] (2007). Methodologies to diagnose and monitor dry eye disease: Report of the Diagnostic Methodology Subcommittee of the International Dry Eye WorkShop (2007). Ocul. Surf..

[54] Dai Y.-P., Duan Y., Lu Y.-T., Ni X.-T., Zhang Y.-K., Li J., Li S.-X. (2024). Nourishing Yin traditional Chinese medicine: Potential role in the prevention and treatment of type 2 diabetes. Am. J. Transl. Res..

[55] Bron A.J., Evans V.E., Smith J.A. (2003). Grading Of Corneal and Conjunctival Staining in the Context of Other Dry Eye Tests. Cornea.

[56] Kaercher T., Bron A.J. (2008). Classification and Diagnosis of Dry Eye. Surgery for the Dry Eye: Scientific Evidence and Guidelines for the Clinical Management of Dry Eye Associated Ocular Surface Disease.

[57] Li L., Jia Q., Wang Y., Wang X., Ling J. (2021). Effect of Chaihu Shugansan on Mitochondrial Function and Mitophagy in Gastric Tissue of Rats with Functional Dyspepsia. Chin. J. Exp. Tradit. Med. Formulae.

[58] Yang Q., Xu Y., Feng G., Hu C., Zhang Y., Cheng S., Zhang X., Yang Y. (2014). p38 MAPK signal pathway involved in anti-inflammatory effect of Chaihu-Shugan-San and Shen-ling-bai-zhu-San on hepatocyte in non-alcoholic steatohepatitis rats. Afr. J. Tradit. Complement. Altern. Med..

[59] Zhu L., Shen J., Zhang C., Park C.Y., Kohanim S., Yew M., Parker J.S., Roy S. (2009). Chuck Inflammatory cytokine expression on the ocular surface in the Botulium toxin B induced murine dry eye model. Mol. Vis..

[60] Tsubota K., Kaido M., Yagi Y., Fujihara T., Shimmura S. (1999). Diseases associated with ocular surface abnormalities: The importance of reflex tearing. Br. J. Ophthalmol..

[61] Yeh T.N., Graham A.D., Lin M.C. (2015). Relationships among Tear Film Stability, Osmolarity, and Dryness Symptoms. Optom. Vis. Sci..

