# Supplementary material for: Effects of Traditional Chinese Medicine for Prevention and Management of Dry Eye Disease: A Systematic Review, Network Meta-Analysis and Meta-Regression of Randomized Controlled Trials
Source: Vision (Basel). 2026 Oct 6;10(4):76. doi: 10.3390/vision10040076 (PMC13709822; doi:10.3390/vision10040076)

Supplementary Materials: Effects of Traditional Chinese Medicine for Prevention and Management of Dry Eye Disease: A Systematic Review, Network Meta-Analysis and Meta-Regression of Randomized Controlled Trials

Weili Hu, Yuqing Jiang, Man Sau Wong and Liping Zhou

Table S1. Search Strings and Limits for Each Database

Database	Keywords / Search Query
**PubMed**	(("Drugs, Chinese Herbal"[Mesh] OR "Chinese Herbal Drugs" OR "Medicine, Chinese Traditional"[Mesh] OR "Phytotherapy"[Mesh] OR "Chinese Plant Extracts" OR Lycium OR Rehmannia OR Astragalus OR Ophiopogon OR Qiming) AND ("Dry Eye Syndromes"[Mesh] OR "dry eye" OR "keratoconjunctivitis sicca" OR "meibomian gland dysfunction" OR TBUT OR "Schirmer I test" OR "corneal fluorescein staining" OR OSDI) AND (randomized controlled trial[pt] OR randomized[tiab]) AND english[lang] AND Humans[Mesh])
**CINAHL**	(MH "Herbal Medicine+" OR MH "Chinese Medicine+" OR MH "Phytotherapy+" OR "Chinese Herbal Drugs" OR "Traditional Chinese Medicine") AND (MH "Dry Eye Syndrome+" OR "dry eye" OR "keratoconjunctivitis sicca" OR TBUT OR "Schirmer I test" OR "corneal fluorescein staining" OR OSDI) AND (Randomized Controlled Trial) AND English
**Cochrane Library**	("Chinese herbal" OR "traditional Chinese medicine" OR phytotherapy OR Lycium OR Rehmannia OR Astragalus OR Ophiopogon OR Qiming) AND ("dry eye" OR "keratoconjunctivitis sicca" OR "meibomian gland dysfunction" OR TBUT OR "Schirmer I test" OR fluorescein) IN Trials
**Embase**	('traditional chinese medicine'/exp OR 'chinese herbal drug' OR 'phytotherapy'/exp OR lycium:ti,ab,kw OR rehmannia:ti,ab,kw OR astragalus:ti,ab,kw) AND ('dry eye'/exp OR 'dry eye disease':ti,ab,kw OR 'keratoconjunctivitis sicca'/exp OR tbut:ti,ab,kw OR 'schirmer i test':ti,ab,kw OR 'corneal fluorescein staining':ti,ab,kw) AND 'randomized controlled trial'/de AND [english]/lim AND [human]/lim
**CNKI**	((Traditional Chinese medicine (中药) OR Chinese herbal medicine (中草药) OR traditional Chinese medicine (中医药) OR Chinese patent medicine (中成药) OR Qi Ju Di Huang Wan (杞菊地黄丸) OR Qiming Granules (杞明颗粒) OR Astragalus (黄芪) OR Rehmannia (地黄) OR Goji berry/Lycium barbarum (枸杞) OR Ophiopogon japonicus (麦冬)) AND (dry eye (干眼) OR dry eye disease (干眼症) OR keratoconjunctivitis sicca (干燥性角结膜炎) OR tear film break-up time (泪膜破裂时间) OR TBUT OR Schirmer I test (Schirmer I 试验) OR corneal fluorescein staining (角膜荧光素染色)) AND (randomized controlled trial (随机对照试验)).

Table S2. Details for Network Diagram (SIT)

Treatment	Number of Direct Comparisons	Number of Subjects
Heat-Clearing vs. Oral Placebo	1	238
Heat-Clearing vs. Standard care only	1	162
Liver-Spleen Regulating vs. Standard care only	2	272
Liver-Spleen Regulating vs. Topical Placebo	1	60
Modern vs. Oral Placebo	1	50
Oral Placebo vs. Qi-Yin Support	1	60
Oral Placebo vs. Yin-Kidney	3	230
Qi-Yin Support vs. Standard care only	2	124
Standard care only vs. Yin-Kidney	12	1728
Standard care only vs. Yin-Moistening	1	120
Topical Placebo vs. Yin-Kidney	1	106
Topical Placebo vs. Yin-Moistening	2	184

Table S3. Details for Network Diagram (TBUT)

Treatment	Number of Direct Comparisons	Number of Subjects
Heat-Clearing vs. Oral Placebo	1	238
Heat-Clearing vs. Standard care only	2	222
Liver-Spleen Regulating vs. Standard care only	2	272
Liver-Spleen Regulating vs. Topical Placebo	1	60
Qi-Yin Support vs. Standard care only	1	80
Standard care only vs. Yin-Kidney	12	1638
Standard care only vs. Yin-Moistening	1	120
Topical Placebo vs. Yin-Kidney	1	106
Topical Placebo vs. Yin-Moistening	2	184

Table S4. Details for Network Diagram (FL)

Treatment	Number of Direct Comparisons	Number of Subjects
Heat-Clearing vs. Oral Placebo	1	238
Heat-Clearing vs. Standard care only	1	162
Liver-Spleen Regulating vs. Standard care only	1	212
Liver-Spleen Regulating vs. Topical Placebo	1	60
Oral Placebo vs. Yin-Kidney	1	50
Qi-Yin Support vs. Standard care only	1	160
Standard care only vs. Yin-Kidney	5	802
Standard care only vs. Yin-Moistening	1	120
Topical Placebo vs. Yin-Moistening	1	60

Table S5. SIT League Table

	Qi-Yin Support	Yin-Kidney	Modern	Yin-Moistening	Liver-Spleen Regulating	Heat-Clearing	Topical Placebo	Standard care only
**Yin-Kidney**	2.76 (-2.23 to 7.84)							
**Modern**	2.18 (-8.35 to 12.66)	-0.58 (-10.52 to 9.22)						
**Yin-Moistening**	2.95 (-4.56 to 10.44)	0.19 (-5.86 to 6.29)	0.77 (-10.63 to 12.27)					
**Liver-Spleen Regulating**	3.66 (-3.07 to 10.49)	0.90 (-4.43 to 6.26)	1.48 (-9.61 to 12.66)	0.71 (-6.25 to 7.57)				
**Heat-Clearing**	5.13 (-2.58 to 12.81)	2.37 (-4.32 to 8.96)	2.95 (-8.38 to 14.32)	2.18 (-6.59 to 10.90)	1.47 (-6.64 to 9.64)			
**Topical Placebo**	5.15 (-2.05 to 12.34)	2.39 (-3.15 to 7.92)	2.97 (-8.19 to 14.21)	2.20 (-2.79 to 7.04)	1.49 (-4.45 to 7.49)	0.02 (-8.29 to 8.38)		
**Standard care only**	**5.32 (0.63 to 10.06)**	**2.56 (0.25 to 4.86)**	3.14 (-6.75 to 13.21)	2.37 (-3.51 to 8.21)	1.66 (-3.32 to 6.59)	0.19 (-6.20 to 6.68)	0.17 (-5.30 to 5.63)	
**Oral Placebo**	**6.18 (0.83 to 11.61)**	3.42 (-0.54 to 7.39)	4.00 (-5.03 to 13.01)	3.23 (-3.91 to 10.43)	2.52 (-4.00 to 9.00)	1.05 (-5.75 to 7.97)	1.03 (-5.64 to 7.74)	0.86 (-3.36 to 5.15)

Table S6. TBUT League Table

	Yin-Moistening	Liver-Spleen Regulating	Yin-Kidney	Qi-Yin Support	Heat-Clearing	Modern	Oral Placebo	Topical Placebo
**Liver-Spleen Regulating**	0.39 (-1.80 to 2.68)							
**Yin-Kidney**	0.45 (-1.50 to 2.41)	0.06 (-1.70 to 1.77)						
**Qi-Yin Support**	0.38 (-3.35 to 4.13)	-0.01 (-3.60 to 3.53)	-0.07 (-3.44 to 3.28)					
**Heat-Clearing**	1.13 (-1.56 to 3.78)	0.74 (-1.75 to 3.12)	0.68 (-1.45 to 2.76)	0.75 (-3.01 to 4.47)				
**Modern**	1.25 (-2.03 to 4.59)	0.87 (-2.29 to 3.97)	0.80 (-2.08 to 3.70)	0.88 (-3.33 to 5.11)	0.12 (-3.20 to 3.47)			
**Oral Placebo**	1.48 (-2.18 to 5.09)	1.10 (-2.43 to 4.49)	1.03 (-2.23 to 4.22)	1.11 (-3.37 to 5.58)	0.35 (-2.09 to 2.82)	0.23 (-3.94 to 4.31)		
**Topical Placebo**	**1.80 (0.29 to 3.28)**	1.41 (-0.76 to 3.50)	1.34 (-0.55 to 3.23)	1.42 (-2.32 to 5.15)	0.67 (-1.98 to 3.32)	0.54 (-2.74 to 3.83)	0.31 (-3.31 to 3.94)	
**Standard care only**	**2.49 (0.64 to 4.37)**	**2.10 (0.57 to 3.59)**	**2.04 (1.12 to 2.98)**	2.11 (-1.10 to 5.33)	1.36 (-0.53 to 3.31)	1.24 (-1.47 to 3.97)	1.01 (-2.07 to 4.14)	0.69 (-1.15 to 2.58)

Table S7. FL League Table (SMD)

	**Yin-Kidney**	**Liver-Spleen Regulating**	**Heat-Clearing**	**Yin-Moistening**	**Standard care only**	**Oral Placebo**	**Topical Placebo**
**Liver-Spleen Regulating**	0.06 (-0.72 to 0.83)						
**Heat-Clearing**	0.21 (-0.47 to 0.94)	0.14 (-0.78 to 1.13)					
**Yin-Moistening**	0.29 (-0.51 to 1.07)	0.22 (-0.61 to 1.05)	0.08 (-0.92 to 1.02)				
**Standard care only**	**0.37 (0.00 to 0.71)**	0.30 (-0.39 to 0.99)	0.16 (-0.54 to 0.79)	0.08 (-0.63 to 0.78)			
**Oral Placebo**	0.50 (-0.15 to 1.28)	0.43 (-0.50 to 1.51)	0.29 (-0.32 to 1.00)	0.21 (-0.74 to 1.29)	0.13 (-0.53 to 0.94)		
**Topical Placebo**	0.65 (-0.26 to 1.55)	0.58 (-0.16 to 1.33)	0.44 (-0.66 to 1.48)	0.36 (-0.39 to 1.11)	0.28 (-0.55 to 1.12)	0.15 (-1.03 to 1.19)	
**Qi-Yin Support**	0.77 (-0.10 to 1.64)	0.70 (-0.34 to 1.76)	0.56 (-0.51 to 1.57)	0.48 (-0.58 to 1.55)	0.40 (-0.39 to 1.20)	0.27 (-0.89 to 1.28)	0.12 (-1.03 to 1.27)

Table S8. Clinical Characteristics and Methodological Features of Included Studies (n = 33)

Author (Year)	Node	Severity	Route	Design
Chang, Lin (2005) [16]	Yin-Kidney	U	O	A
Chen, Chen (2014) [26]	Yin-Kidney	U	O	A
Chen, Chen (2015) [25]	Yin-Kidney	P	O	A
Chen, Wang (2006) [31]	Qi-Yin Support	P	O	A
Fan, Hung (2024) [21]	Yin-Kidney	S	O	M
Gao, Chen (2022) [37]	Yin-Kidney	P	O	A
Han, Dong (2023) [41]	Qi-Yin Support	S	O	M
Heng (2020) [29]	Yin-Kidney	S	O	A
Hu, Qin (2018) [46]	Heat-Clearing	P	O	A
Hui, Ni (2022) [28]	Liver-Spleen Regulating	S	O	M
Kan, Wang (2020) [15]	Modern	U	O	M
Leng (2018) [35]	Yin-Kidney	U	O	A
Lin, Pang (2020) [47]	Heat-Clearing	U	T	A
Liu, Jin (2020) [19]	Heat-Clearing	U	T	M
Long (2014) [44]	Yin-Kidney	U	O	A
Nagaki (2003) [18]	Yin-Kidney	U	O	M
Ou, Wang (2025) [17]	Heat-Clearing	U	O	M
Pu (2010) [38]	Yin-Kidney	U	O	M
Rolando (2007) [20]	Modern	U	T	M
Song (2013) [42]	Liver-Spleen Regulating	S	O	M
Tao, Chen (2016) [24]	Yin-Moistening	U	O	A
Wang, Li (2011) [30]	Yin-Moistening	U	O	M
Xia, Yang (2018) [36]	Yin-Kidney	U	O	M
Xiang, Zhang (2011) [45]	Yin-Kidney	U	O	A
Xie, Luo (2018) [32]	Liver-Spleen Regulating	U	O	A
Xu, Ye (2010) [43]	Yin-Kidney	S	O	A
Yang, Zhang (2014) [39]	Yin-Kidney	S	O	A
Yi, Zhang (2016) [33]	Yin-Kidney	U	O	A
Yu, Zeng (2012) [34]	Yin-Kidney	S	O	A

Zeng, Xia (2014) New Chinese Medicine [27]	Liver-Spleen Regulating	P	O	A
Zeng, Xia (2014) [23]	Yin-Kidney	P	O	A
Zhang, Wang (2015) [40]	Qi-Yin Support	S	O	A
Zhao (2017) [22]	Yin-Moistening	P	C	M

Legend: Severity: U = uncomplicated; S = severe; P = post-surgical.

Route: O = oral; T = topical; C = combined (oral + topical).

Design: M = monotherapy; A = add-on.

Table S9. Classification framework for traditional Chinese medicine interventions: five pattern-based classes and the Modern node

Class (abbreviation)	Definition
**Yin–Kidney (YK)**	Tonifies the Liver and Kidney and replenishes essence ("nourishes the water wheel"); indicated for age-related decline and essence–blood depletion with inadequate ocular-surface and fundus lubrication. Dominated by kidney-tonifying, essence-replenishing herbs such as Rehmannia (Shudihuang), Cornus (Shanzhuyu), Lycium (Gouqizi), Cuscuta (Tusizi), and Rubus (Fupenzi).
**Liver–Spleen Regulating (LSR)**	Soothes Liver qi, strengthens the Spleen, and regulates qi to transport fluids; indicated for emotional disturbance or impaired splenic transportation with fluids failing to ascend. Dominated by qi-regulating, spleen-fortifying, dampness-resolving herbs such as Bupleurum (Chaihu), Paeonia (Baishao), Atractylodes (Baizhu), and Poria (Fuling).
**Qi–Yin Support (QYS)**	Replenishes qi, nourishes yin, and promotes fluid generation; indicated for dual qi–yin deficiency with qi failing to propel fluids upward. Dominated by qi-tonifying herbs such as Astragalus (Huangqi), Codonopsis (Dangshen), and American ginseng (Xiyangshen), combined with yin-nourishing herbs.
**Yin–Moistening (YM)**	Nourishes yin, moistens dryness, and promotes fluid production; indicated for ocular dryness due to fluid depletion failing to ascend. Dominated by yin-nourishing, dryness-moistening herbs such as Ophiopogon (Maidong), Scrophularia (Xuanshen), Polygonatum odoratum (Yuzhu), and Lily (Baihe).
**Heat-Clearing (HC)**	Clears heat, purges fire, and clears the Liver to brighten the eyes; indicated for damp-heat or exuberant Liver-fire dry eye with redness, congestion, and burning pain. Dominated by heat-clearing, eye-brightening herbs such as Chrysanthemum (Juhua), Scutellaria (Huangqin), and Prunella (Xiakucao).
**Modern**	Strictly limited to single compounds or modern chemically purified agents without classical pattern-based formulation (e.g., the lutein formula of Kan & Wang 2020 and tamarind seed

	polysaccharide of Rolando 2007), thereby clearly distinguished from traditional decoctions and patent medicines.

Figure S1. Meta-regression of mean age versus change in (A) SIT, (B) TBUT, and (C) FL, respectively

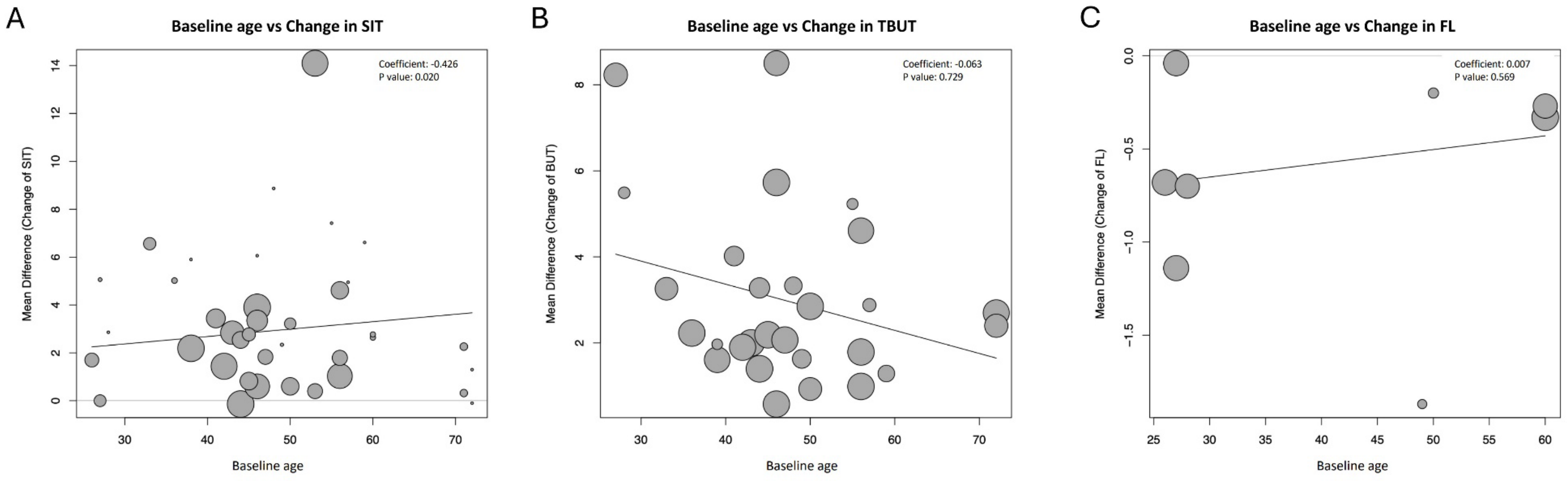

Figure S2. Meta-regression of duration (weeks) versus change in (A) SIT, (B) TBUT, and (C) FL, respectively

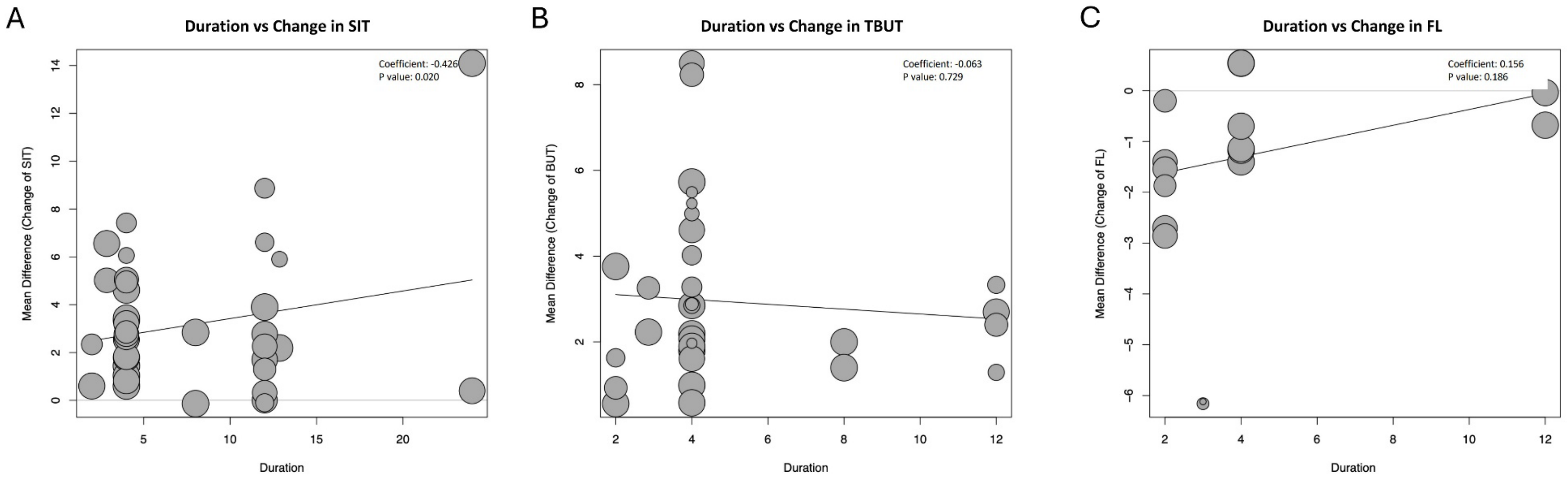

Figure S3. Heterogeneity Assessment with respect to potential effect modifiers: (A)Age; (B) Duration (weeks); (C) Gender (% male); (D) Sample Size

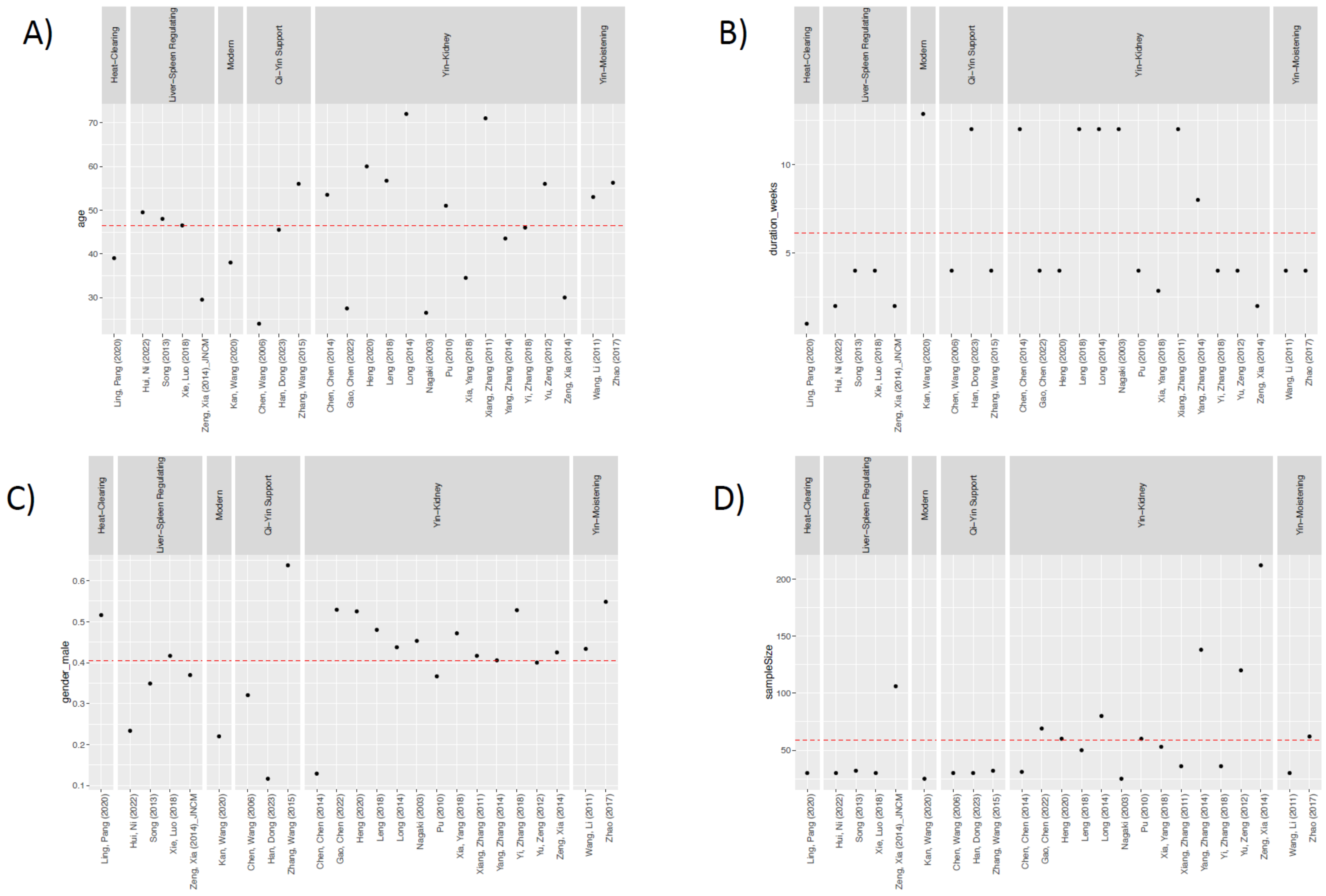

Figure S4. Risk of bias proportion graph (as percentage) for all included randomized controlled trials.

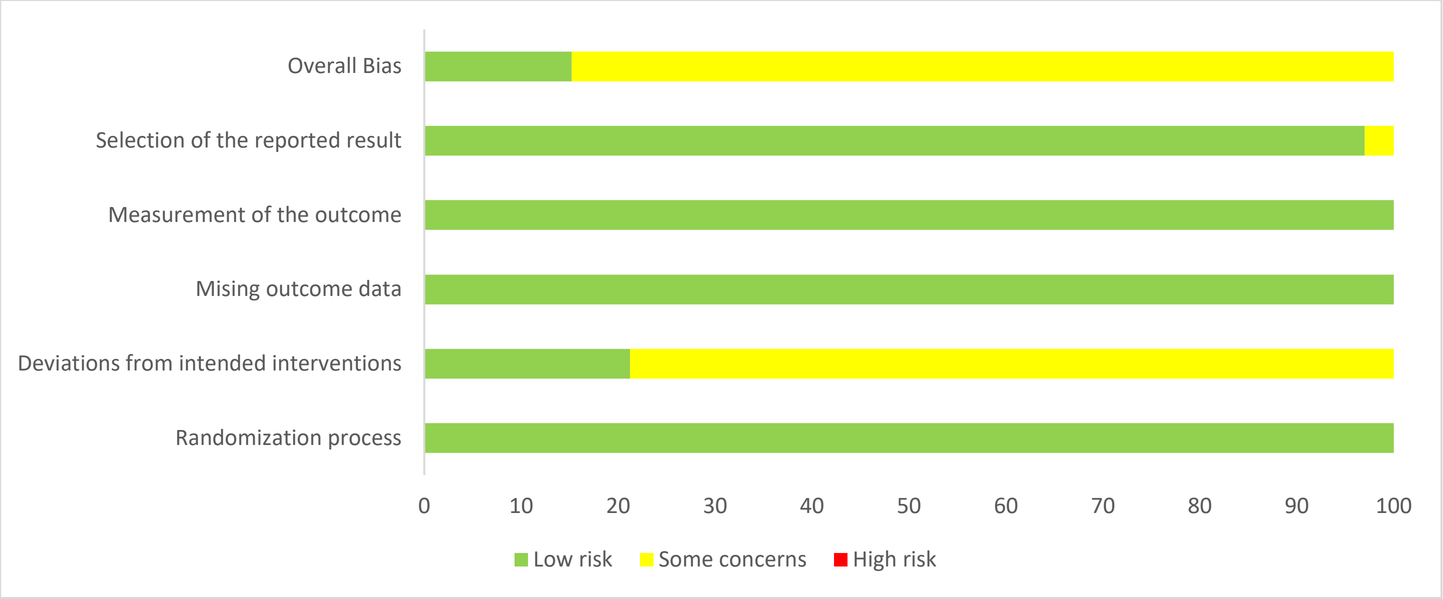

Supplement: Supplementary file 1 [file vision-10-00076-s001.zip › vision-4540290-supplementary.pdf]
